# A densely interconnected network for deep learning accelerated MRI

**DOI:** 10.1007/s10334-022-01041-3

**Published:** 2022-09-14

**Authors:** Jon André Ottesen, Matthan W. A. Caan, Inge Rasmus Groote, Atle Bjørnerud

**Affiliations:** 1 Computational Radiology & Artificial Intelligence (CRAI) Unit, Division of Radiology and Nuclear Medicine, Oslo University Hospital, Oslo, Norway; 2 Department of Physics, Faculty of Mathematics and Natural Sciences, University of Oslo, Oslo, Norway; 3 Amsterdam UMC, Biomedical Engineering and Physics, University of Amsterdam, Amsterdam, Netherlands; 4 Department of Radiology, Vestfold Hospital Trust, Tønsberg, Norway

**Keywords:** MRI, Deep learning, Image reconstruction

## Abstract

**Objective:**

To improve accelerated MRI reconstruction through a densely connected cascading deep learning reconstruction framework.

**Materials and methods:**

A cascading deep learning reconstruction framework (reference model) was modified by applying three architectural modifications: input-level dense connections between cascade inputs and outputs, an improved deep learning sub-network, and long-range skip-connections between subsequent deep learning networks. An ablation study was performed, where five model configurations were trained on the NYU fastMRI neuro dataset with an end-to-end scheme conjunct on four- and eightfold acceleration. The trained models were evaluated by comparing their respective structural similarity index measure (SSIM), normalized mean square error (NMSE), and peak signal to noise ratio (PSNR).

**Results:**

The proposed densely interconnected residual cascading network (DIRCN), utilizing all three suggested modifications achieved a SSIM improvement of 8% and 11%, a NMSE improvement of 14% and 23%, and a PSNR improvement of 2% and 3% for four- and eightfold acceleration, respectively. In an ablation study, the individual architectural modifications all contributed to this improvement for both acceleration factors, by improving the SSIM, NMSE, and PSNR with approximately 2–4%, 4–9%, and 0.5–1%, respectively.

**Conclusion:**

The proposed architectural modifications allow for simple adjustments on an already existing cascading framework to further improve the resulting reconstructions.

## Introduction

Magnetic resonance imaging (MRI) data acquisition is an inherently slow process due to fundamental physical constraints that limit the rate of k-space traversal. This can lead to prolonged MRI sequences, during which the patient must remain still to achieve images of diagnostic quality. Traditionally, parallel imaging [1–3] and compressed sensing [4] have been used to reduce aliasing artifacts caused by the subsampling of k-space. This allows for the reconstruction of clinically acceptable images with up to twofold acceleration for brain MRI [5].

In recent years, deep learning and convolutional neural networks (CNNs) have shown great promise as an alternative framework for MRI reconstruction to further accelerate scans beyond that of parallel imaging and compressed sensing. A study has shown that the end-to-end variational network [6] can reconstruct images that are interchangeable for the detection of internal derangements of the knee when compared to their fully sampled counterparts at fourfold acceleration [7].

Deep learning MRI reconstruction frameworks span a wide variety of different architectures, from U-Net-based models [8] with [9] and without [10] data consistency in k-space; general adversarial networks [11]; k-space reconstruction networks [12]; cascaded networks that consisting of sub-networks for temporal dynamic acquisitions [13–15] and non-temporal static acquisitions [6, 16–20], in which the latter performs excellently [21, 22]. The sub-networks perform reconstruction in the image domain, frequency domain or both, and reconstruct the complete image or frequency information from the subsampled scan. CNNs are commonly used as sub-networks, and the architectures range from shallow sub-networks tallying a few convolutional layers per cascade [18, 23], to deeper architectures [6, 16, 24].

In this work, we sought to improve the overall reconstruction quality for cascading networks for static image acquisition, by introducing and testing three novel improvements. The end-to-end variational network [6] was adopted as a reference model, and from this, we developed the densely interconnected residual cascading network (DIRCN). The contributions of DIRCN are summarized as follows:

1. Input level dense connections [25]were implemented to improve gradient and information flow through cascades in a similar manner to previous implementations [18, 26].
2. A U-Net-based sub-network that incorporates aggregated [27] residual connections [28] with squeeze-and-excitation [29], and the sigmoid linear unit (SiLU) activation function [30–32] was adopted to improve in-cascade gradient flow and expressivity through channel-wise excitation.
3. Long range skip-connections across sub-networks were implemented; we hypothesized that long-range skip-connections will further improve gradient flow and further fine-tune feature maps.

Focus was placed on facilitating gradient flow and connectivity between sub-networks.

The architectural modifications proposed in this study were tested on the NYU fastMRI neuro dataset [33, 34] to gauge the importance of the input-level dense connections, long-range skip-connections, and the proposed U-Net-based sub-network for four- and eightfold k-space subsampling.

## Methods

This section provides an overview of the problem formulation, network architecture, dataset, training scheme, and model evaluation. Additional details regarding the model implementation are given in the source repository.^1^

### Problem formulation

For 2D cartesian acquisition, let $k\in {\mathbb{C}}^{c\times {n}_{kx}\times {n}_{ky}}$ denote the fully sampled multi-coil complex-valued k-space representation for $c$ receiver coils with ${n}_{kx}$ and ${n}_{ky}$ sampled datapoints along the frequency and phase-encoding dimensions, respectively. The corresponding image representation $x\in {\mathbb{C}}^{c\times {n}_{kx}\times {n}_{ky}}$ of the sampled k-space for the j-th coil element is related by

1 $$k_{j} = F\left( {S_{j} \, \circ \, x_{j} } \right) + \varepsilon,$$

where $\mathcal{F}$ is the two-dimensional Fourier transform, ${S}_{j}$ is the coil sensitivity for the j-th receiver coil, $\circ$ is the Hadamard product (element-wise multiplication), and $\upepsilon$ is additive noise.

The speed by which k-space is traversed is governed by the number of phase-encoding steps ${n}_{ky}$. To accelerate MRI acquisition, k-space can be subsampled by reducing the number of phase-encoding steps. From the fully sampled k-space data, $k$, the subsampled subset of k-space is given by

2 $$k_{u} = U \, \circ \, k,$$

where ${k}_{u}\in {\mathbb{C}}^{c\times {n}_{kx}\times {n}_{ky}}$ is the undersampled k-space and $U\in {\mathbb{C}}^{{n}_{kx}\times {n}_{ky}}$ is a binary undersampling mask. The acceleration factor is the ratio between the number of masked lines and the total number of acquired lines.

The intention of image reconstruction is to solve the inverse problem of recovering the image representation $x$ from the undersampled k-space, ${k}_{u}$. To that end, supervised deep learning networks aim to map a subsampled k-space to the corresponding fully sampled k-space by learning from pairs of undersampled and fully sampled scans.

### Network architecture

**Fig. 1:**
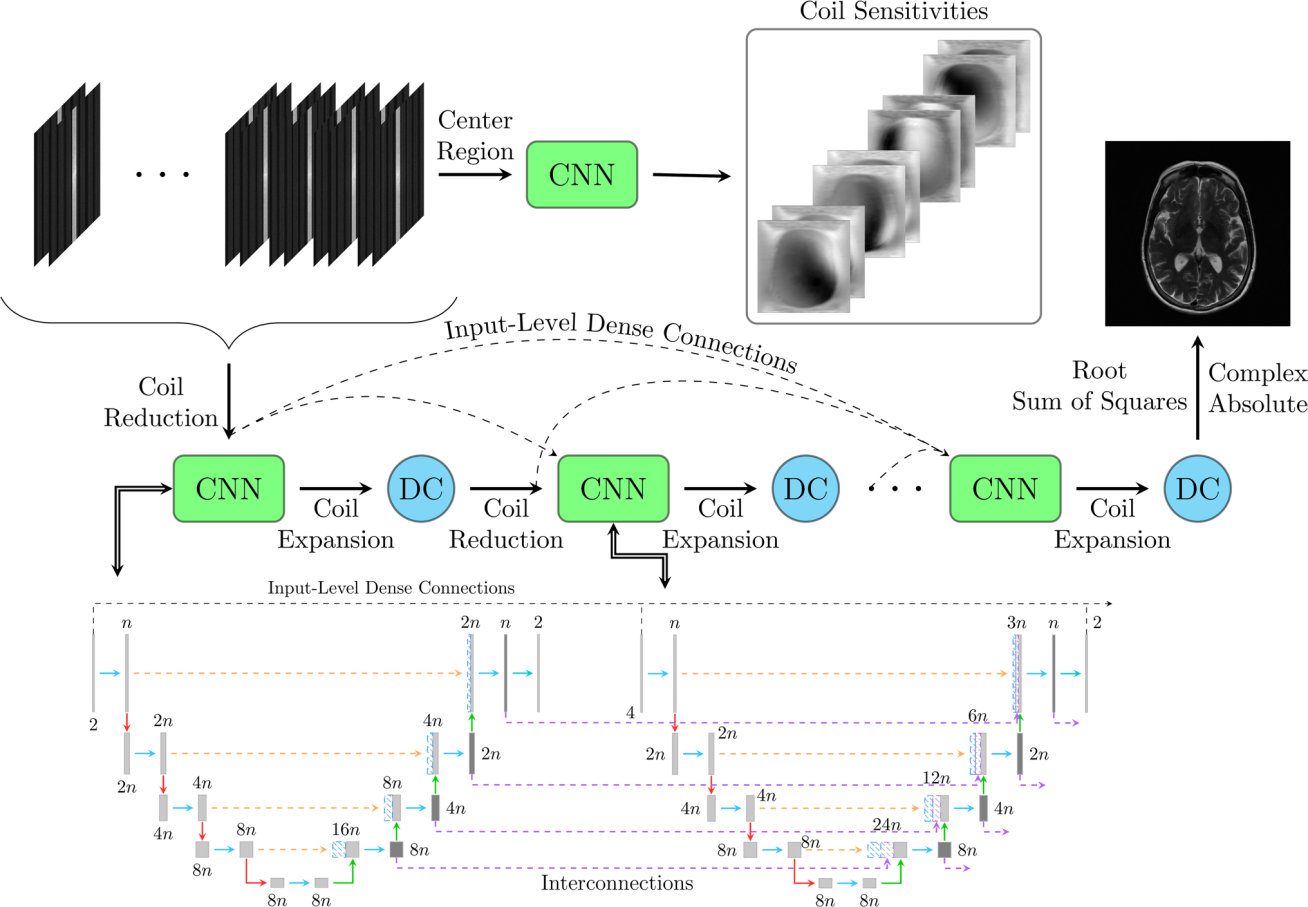
An illustration of the dense interconnected residual cascading network (DIRCN). The model consists of *m* cascades of a simplified U-Net-based architecture and data consistency (DC). Each cascade is connected to every prior cascade by input-level dense connections illustrated by the black dashed lines. Every sub-network is connected to the prior sub-network through concatenation, dubbed interconnections and they are illustrated by the purple dashed lines. The output is the root-sum-of-squares image of the data-consistent output from the last cascade

This work presents a densely interconnected residual cascading network (DIRCN) for MRI reconstruction. DIRCN builds on top of the end-to-end variational network [6], and the end-to-end variational network was adopted as the reference model and was used to benchmark performance. The novelty in this work stems from the three extensions employed to this reference model. With these modifications, we sought to improve the gradient flow and connectivity between the cascading layers. To that end, the reference model was extended by: (1) Long range dense input-level connections, (2) a U-Net-based CNN sub-network, and (3) long-range skip-connections dubbed interconnections. The general DIRCN model architecture is illustrated in Fig. 1, where the U-Net-based CNN sub-network is replaced by a simplified CNN for readability.

### Reference model

The reference network follows the end-to-end variational network [6], but the variational update mechanism implemented by Hammernik et al. [35] was changed to a data consistency method similar to Schlemper et al. [18]. Given a set of subsampled k-space, ${k}_{u}$, and a corresponding k-space prediction, ${k}_{p}$, data consistency was be implemented by

3 $$f_{{{\text{dc}}}} \left( {k_{p} } \right) = \left\{ {\begin{array}{*{20}c} {\frac{{k_{u} + \lambda \times k_{p} }}{1 + \lambda }} & {U_{i,j} = 1} \\ {k_{p} } & {U_{i,j} = 0} \\ \end{array} } \right.,$$

where $\lambda$ is a learnable parameter initialized to $0.01$.

Given an undersampled k-space sample, ${k}_{u}$, the coil sensitivities, $S$, were estimated identical to the reference model with a CNN using the fully sampled center portion of k-space. Note that the network architecture used for coil sensitivity estimation was identical to the sub-networks embedded for image reconstruction, except a lower number of parameters to reduce memory constraints.

The reference model consists of $m$ cascades, each of which consists of a series of five distinct operations:

1. The coil sensitivities, ${S}_{i}$, are estimated by a separate model with the same architecture as the sub-network, with two channels: one for the real and imaginary component, respectively. The batch dimension is used for each coil-element, this allows for a varying coil dimensionality and the coil sensitivities are normalized ${\sum }_{i}\overline{{S}_{i}}{S}_{i}=1$ to satisfy the constraints detailed by [36].^2^
2. The coil dimensionality for the k-space representation, ${k}_{u}$, is reduced by ${I}_{\mathrm{red}}={\sum }_{i=1}^{{n}_{c}}{\mathcal{F}}^{-1}\left({k}_{u}^{i}\right)\overline{{S}_{i}}$, effectively reducing the number of channels from c-coils to a single complex image.
3. The coil reduced image is refined by a CNN: ${I}_{\mathrm{rec}}=\mathrm{CNN}\left({I}_{\mathrm{red}}\right)$, where ${I}_{\mathrm{rec}}$ is the refined complex coil reduced image. Complex valued CNN-inputs were handled using two channels: one for the real and imaginary component, respectively.
4. The number of coils in the refined image is expanded back to the original number using the coil sensitivities by${I}_{\mathrm{ep}}=\mathrm{cat}\left({I}_{\mathrm{rec}}\circ {S}_{1}, {I}_{\mathrm{rec}}\circ {S}_{2}, ...,{I}_{\mathrm{rec}}\circ {S}_{{n}_{c}-1}, {I}_{\mathrm{rec}}\circ {S}_{{n}_{c}}\right)$, where the *cat* operation is concatenation along the channel dimension.
5. Data consistency is enforced, and the data consistent k-space is given by ${k}_{\mathrm{dc}}={f}_{\mathrm{dc}}\left(\mathcal{F}\left({I}_{\mathrm{ep}}\right)\right)$, where ${f}_{dc}$ is given in Eq. 3.

The cascade output, ${k}_{\mathrm{dc}}$, was used as the input for the next cascade instead of ${k}_{u}$. In this work, the number of cascades was enforced to $m=12$ for all model configurations. The magnitude image was computed by taking the complex absolute followed by the root sum of squares (RSS).

### Input-level dense connections

As a first extension, input-level dense connections [25] were implemented to facilitate gradient and information flow throughout the network. For the k’th cascade, the CNN refinement from step 2 can be written as ${I}_{\mathrm{rec}}^{k}={\mathrm{CNN}}^{k}\left({I}_{\mathrm{red}}^{k}\right)$.^3^ In the case of input-level dense connections, the CNN input is given by the concatenated coil reduced image from the prior cascades. The CNN refinement step for input-level dense connections is given by

4 $$I_{{{\text{rec}}}}^{k} = {\text{CNN}}^{k} \left( {{\text{cat}}\left( {I_{{{\text{red}}}}^{k} ,I_{{{\text{red}}}}^{k - 1} ,...I_{{{\text{red}}}}^{2} ,I_{{{\text{red}}}}^{1} ,} \right)} \right)$$

and the input-level dense connections are illustrated in Fig. 1.

### Refinement of CNNs: ResXUnet

**Fig. 2:**
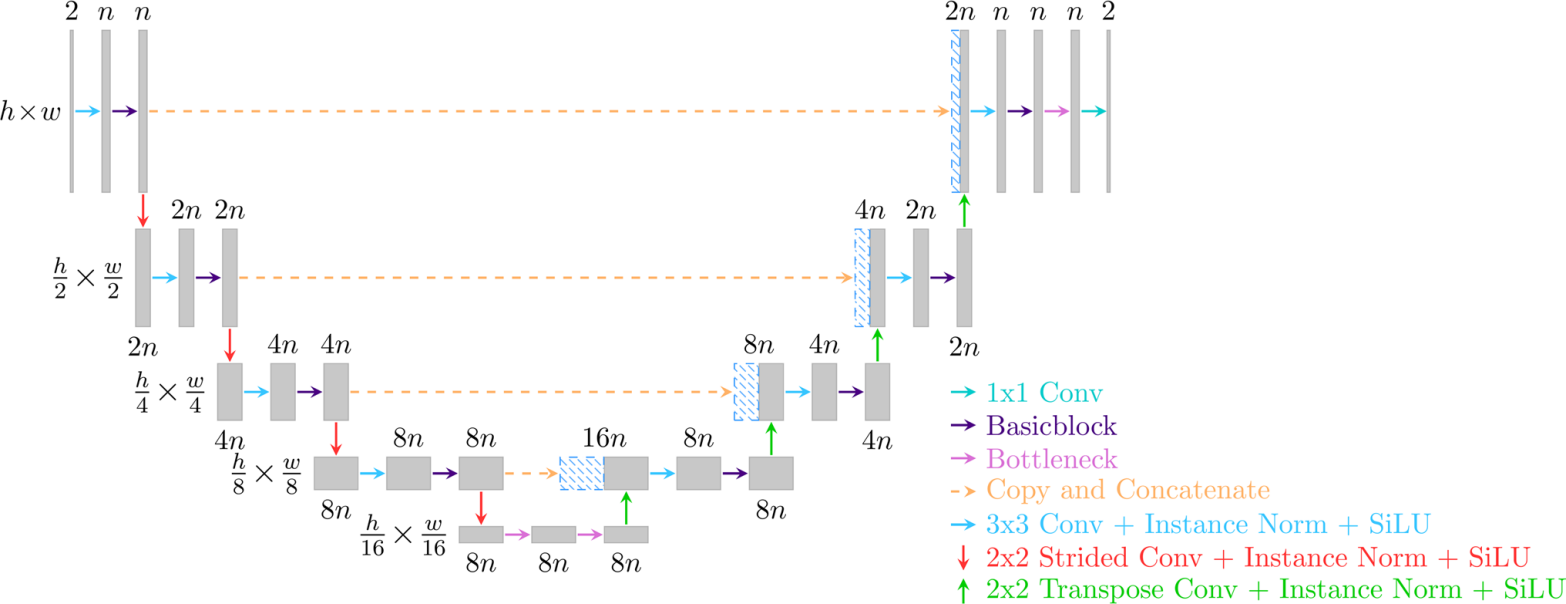
The suggested ResXUNet model used for the CNN refinement step. The model includes aggregated residual connections [27] for improved gradient flow, squeeze-and-excitation [29] for learnable channel-wise attention and the SiLU activation function [30–32]. The squeeze-and-excitation operation was implemented as the final operation in all residual blocks

The second extension originates from the multiple alterations and refinements which have been proposed based on the U-Net [37]. These U-Net alterations utilize different architectural modifications such as residual connections, dense connections, attention mechanisms, and multilayer feature fusion, among others. In this work, a modified U-Net-based model dubbed ResXUNet was embedded into the cascaded network, incorporating aggregated residual connections, squeeze-and-excitation, the SiLU activation function that has shown improved performance over other activation functions [30–32], and instance normalization [38]. The ResXUNet model is illustrated in Fig. 2.

Given a feature map, $x\in {R}^{b, c, h, w}$, residual connections facilitate gradient flow by additive identity mapping

5 $$y = F\left( {x,W_{i} } \right) + x,$$

where $F$ is a set of two (basicblock) or three (bottleneck) convolutional operations with weights ${W}_{i}$, normalization, and non-linear activations [28]. Squeeze-and-excitation model channel-wise dependencies through a global average operation, i.e., a squeeze operation ${x}^{^{\prime}}= {F}_{\mathrm{sq}}(x)$ where ${x}^{^{\prime}}\in {R}^{b, c, 1, 1}$ is the average value for every feature map. The squeeze operation is followed by a learnable excitation operation ${s={\sigma }_{sigmoid}(F}_{\mathrm{ex}}({x}^{^{\prime}}, {W}_{j}))$, where ${\sigma }_{\mathrm{sigmoid}}$ is the sigmoid activation function enforcing $s\in {\left[0, 1\right]}^{b, c, 1, 1}$ and ${F}_{\mathrm{ex}}$ is a feed forward neural network with weights ${W}_{j}$. Channel-wise dependencies are adaptively modeled through a multiplicative channel-wise scaling operation $y= x\cdot s$, i.e., every channel is adaptively recalibrated though a multiplicative scaling operation [29]. As suggested by the authors, the squeeze-and-excitation operation was implemented at the end of every residual block before the identity mapping.

### Long range skip connections—interconnections

The cascading network type can be seen as a series of independent sub-networks, where the input of a sub-network is the data consistent output from a prior sub-network. Besides this connection, each individual sub-network does not share any of the extracted feature maps from a prior sub-network.

To improve the interconnectivity between sub-modules, the third extension is to insert long-range skip connections comparable with those utilized in the U-Net. The interconnections were implemented to connect every subsequent sub-model, thereby creating a flow of feature maps between the sub-networks. This was done by copying the final feature map for each resolution in the deep learning model and concatenating the feature maps for each resolution onto the subsequent sub-network. These connections, coined interconnections, are illustrated in Fig. 1.

### Dataset and undersampling masks

The proposed method was trained, validated, and evaluated on the fully sampled raw k-space fastMRI neuro dataset [33, 34]. The dataset consists of two predetermined splits, one for training with 4469 scans and one for validation with 1378 scans. Both sets consist of T1-weighted pre and post contrast, T2-weighted, and FLAIR images from both 1.5 T and 3 T scanners. The scans have a wide variety of acquisition matrices with and without zero-padding. The predetermined validation set was randomly split up into a test and validation set, with 689 scans in both the validation and test set. The exact distribution used can be found in the source repository.^1^

The fully sampled raw k-space was undersampled by a line-wise equidistant downsampling scheme with a fully sampled center, the masks used in this study is similar to those used in the fastMRI challenge and leaderboard [6, 22, 34]. As such, the center region of k-space and every n’th k-space line were not masked. This scheme was used for both four- and eightfold acceleration, and the center contained 8% or 4% of the number of phase-encoding steps, respectively.

**Fig. 3:**
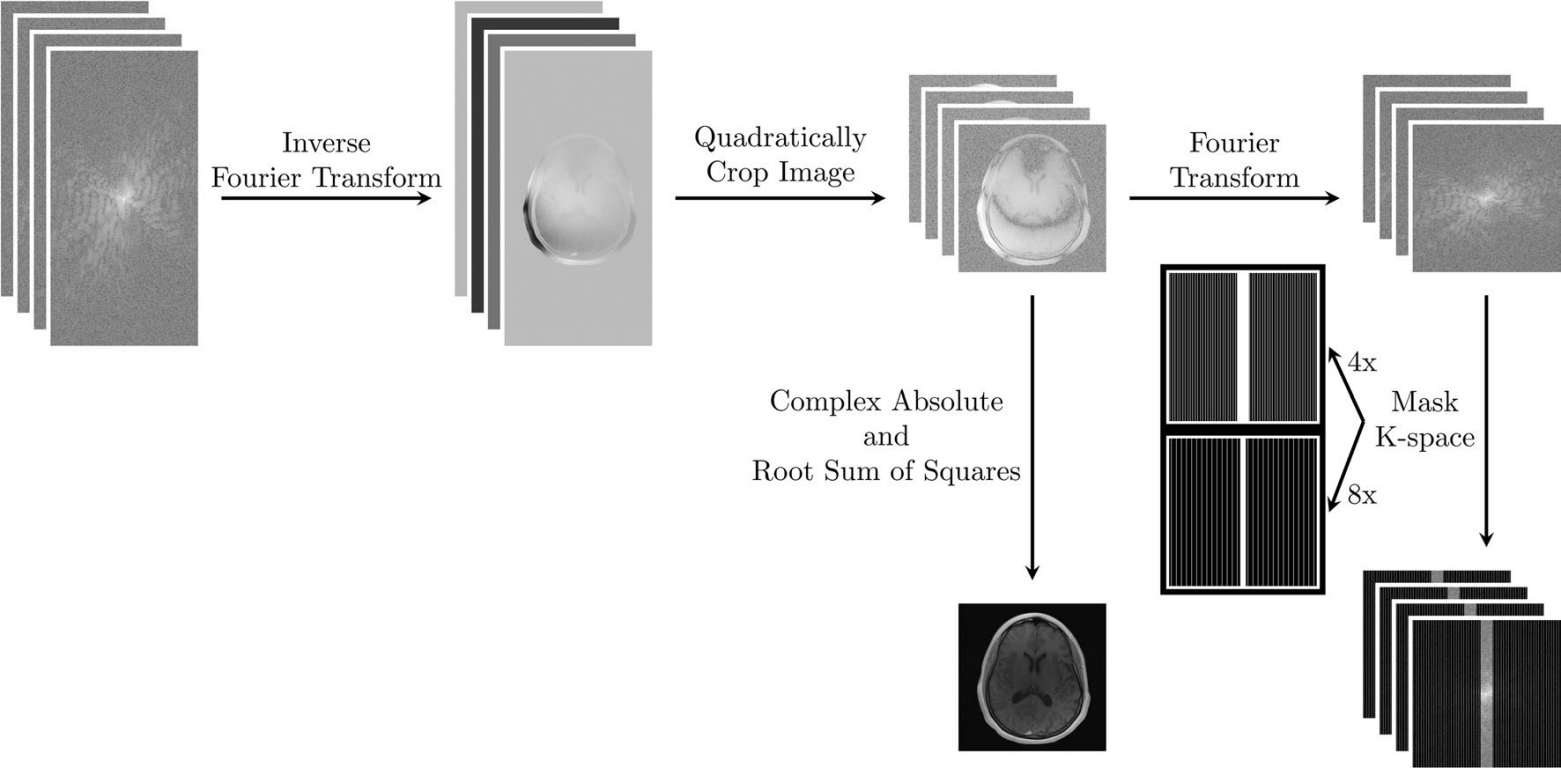
An illustration of the preprocessing steps for the undersampled k-space and the fully sampled magnitude image. Raw multi-coil k-space data were first Fourier transformed to image space, then quadratically cropped along the height and width dimension for all coils. The ground truth image was the complex absolute and root sum of squares of the complex coil images. For model inputs, the cropped complex coil images were Fourier transformed back to k-space before being masked by either a four- or eightfold downsampling mask

To reduce memory requirements, frequency oversampling was removed from the data. This was done by quadratically cropping all images in the image domain, followed by Fourier transforming them back into k-space before being undersampled. The ground truth image is the complex absolute image followed by the RSS than quadratically cropped along the height and width dimension to remove oversampling and emphasize brain voxels. Nonetheless, the model accepts and reconstruct k-space with any arbitrary coil dimensionality and rectangular image size. The preprocessing steps including examples of the equidistant downsampling masks for both acceleration factors are illustrated in Fig. 3.

### Implementation

In total five model configurations were trained. This includes the reference model and DIRCN, after which an ablation study where the three architectural modifications: input-level dense connections, ResXUNet, and interconnections were tested individually. Input-level dense connections and interconnections were in the ablation study tested through modifications of the original U-Net architecture. For the DIRCN, input-level dense connections, and interconnections were implemented in the ResXUNet architecture. The placement of the dense connections and interconnections for the U-Net and ResXUNet architecture corresponds to the illustration in Fig. 1. Coil sensitivity estimation was either performed by the U-Net or ResXUNet architecture, and the architecture used was the same as the network used in the cascades.

All model configurations were trained and implemented in Python using PyTorch version 1.7.1 [39]. The Adam optimizer [40] was used with default PyTorch parameters and an initial learning rate of $0.002$, with stepwise learning-rate decay every 60’th iteration using $\upgamma = 0.1$ and Amsgrad [41] enabled. All models were trained for 120 iterations, with a mini-batch size of one, and every iteration looped over 10,000 randomly selected image slices from the dedicated NYU fastMRI neuro training set. After each iteration, the models were validated on 4,000 randomly selected image slices from the validation set. Each image was undersampled with equal likelihood by either four- or eightfold acceleration during training and validation. Neither data augmentation nor data parallelization was used. The number of parameters was set to approximately 45 million for all model configurations to constrain memory usage for the most memory-intensive models.

Training took approximately ten days for all models not using the ResXUNet architecture and 20 days for the models that used the ResXUNet architecture. All training was done on either a Nvidia V100 (32 GB) or a RTX 3090 (24 GB). All networks were benchmarked on a single RTX 2080 Ti (11 GB). The inference time was computed as the mean of 1000 reconstructions on a single fourfold accelerated slice of size $376\times 376$ with 20 coil elements and randomly initialized model parameters.

The loss function was an equally weighted linear combination of the Gaussian weighted structural similarity index measure (SSIM) and the mean absolute distance (L1 loss). Reconstruction quality was assessed using the SSIM [42], normalized mean square error (NMSE), and peak signal to noise ratio (PSNR). All model configurations were evaluated using the final checkpoint after 120 iterations.

## Results

**Fig. 4:**
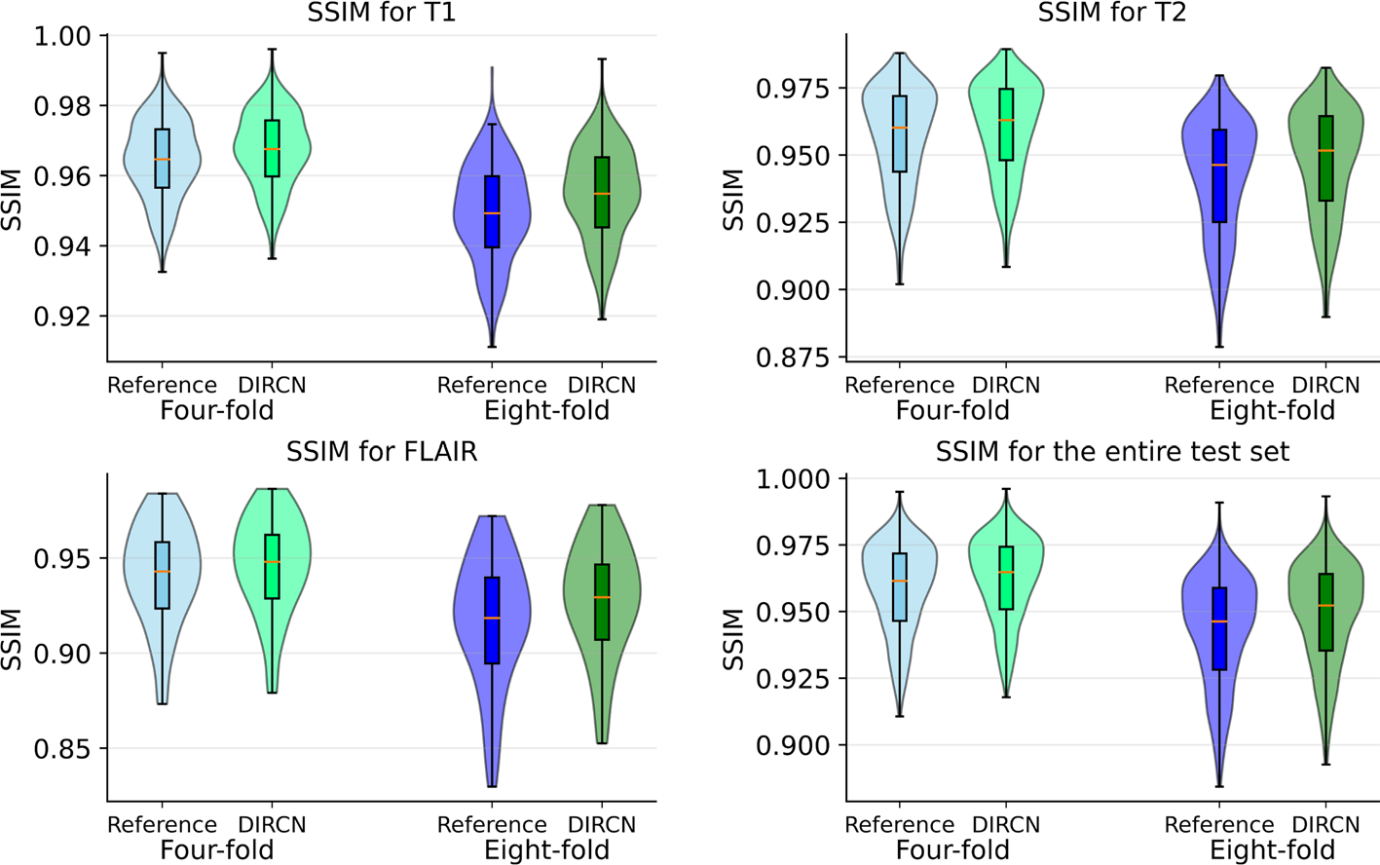
Structural similarity index measure (SSIM) distributions for the designated test dataset for the reference model and DIRCN for T1-weighted, T2-weighted, FLAIR, and all images. The distributions show the SSIM for both four- and eightfold acceleration. Note, alle outlier for low SSIM values are emitted for readability, with the outlier definition following regular conventions

Violin plots of the SSIM-values for the reference model and DIRCN on the test set are shown in Fig. 4. An improved mean SSIM can be observed for all weighting schemes, this effect is more pronounced for eightfold acceleration compared to fourfold acceleration.

The mean SSIM, NMSE, and PSNR for the four- and eightfold accelerated images for the test dataset for the different model configurations are given in Table 1. We see an improvement of 2–4% in SSIM, 5–10% in NMSE, and 0.5–1.0% in PSNR for all suggested improvements for both acceleration factors. The DIRCN achieved a 7.7% and 10.7% improvement in the SSIM, a 14.3% and 23.2% improvement in the NMSE, and a 1.7% and 3.1% improvement in the PSNR for four- and eightfold acceleration, respectively. DIRCN achieved superior PSNR, NMSE, and SSIM compared to the reference model for both four- and eightfold acceleration. DIRCN achieved an improvement in the SSIM, NMSE, and PSNR that is beyond that of the individual improvement for the dense connections, interconnections, and ResXUNet model, individually. E.g., for eightfold acceleration, the NMSE reduced from 0.0088 to 0.0081, 0.0080, and 0081 for dense layers, the ResXUNet and added interconnections, respectively. When all three suggested improvements were jointly in place, the NMSE reduced further to 0.0068. For training, the memory consumption for the different models was approximately 15 GB for the reference model; 15.4 GB for the input-level dense connected model; 16 GB for the interconnected model; 30 GB for the ResXUNet model; and 30 GB for DIRCN.

**Table 1:** The structural similarity index measure (SSIM), normalized mean square error (NMSE), and peak signal to noise ratio (PSNR) for the different architectural modifications for T1-weighted, T2-weighted FLAIR images from the dedicated test dataset for fourfold and eightfold acceleration

Architecture	#Params	Inference time [ms]	Modality	Fourfold acceleration			Eightfold acceleration		
				SSIM	NMSE	PSNR	SSIM	NMSE	PSNR
Reference	45 M	$148\pm 3$	T1	0.9626	0.0033	41.5	0.9466	0.0070	38.2
			T2	0.9556	0.0044	40.0	0.9399	0.0096	36.6
			FLAIR	0.9357	0.0055	39.1	0.9123	0.0111	36.1
			T1 + T2 + FLAIR	*0.9560*	0.0041	40.4	0.9395	0.0088	37.0
Reference + Dense	45 M	$153\pm 2$	T1	0.9637	0.0031	41.7	0.9486	0.0064	38.6
			T2	0.9569	0.0042	40.2	0.9420	0.0085	37.0
			FLAIR	0.9378	0.0053	39.3	0.9154	0.0100	36.5
			T1 + T2 + FLAIR	*0.9574*	0.0039	40.6	0.9417	0.0080	37.5
Reference + ResXUNet	41 M	$394\pm 4$	T1	0.9635	0.0031	41.7	0.9485	0.0065	38.6
			T2	0.9565	0.0042	40.2	0.9417	0.0087	36.9
			FLAIR	0.9370	0.0053	39.3	0.9149	0.0101	36.5
			T1 + T2 + FLAIR	*0.9569*	0.0040	40.6	0.9415	0.0081	37.4
Reference + Interconnections	49 M	$159\pm 2$	T1	0.9638	0.0030	41.8	0.9482	0.0065	38.6
			T2	0.9567	0.0041	40.3	0.9419	0.0085	37.0
			FLAIR	0.9375	0.0053	39.3	0.9144	0.0101	36.5
			T1 + T2 + FLAIR	*0.9573*	0.0039	40.7	0.9415	0.0080	37.5
DIRCN	47 M	$387\pm 5$	T1	0.9658	0.0028	42.2	0.9529	0.0054	39.3
			T2	0.9588	0.0038	40.7	0.9460	0.0072	37.7
			FLAIR	0.9408	0.0048	39.8	0.9216	0.0085	37.2
			**T1 + T2 + FLAIR**	**0.9594**	**0.0035**	**41.1**	**0.9460**	**0.0068**	**38.2**

Bold typefont denote the best performing model for the entire test dataset

Note, for the combined results for all image modalities, all scans were weighted equally although the test dataset had more T1-weighted and T2-weighted compared to FLAIR. The number of parameters and inference time is given for each model configuration

**Fig. 5:**
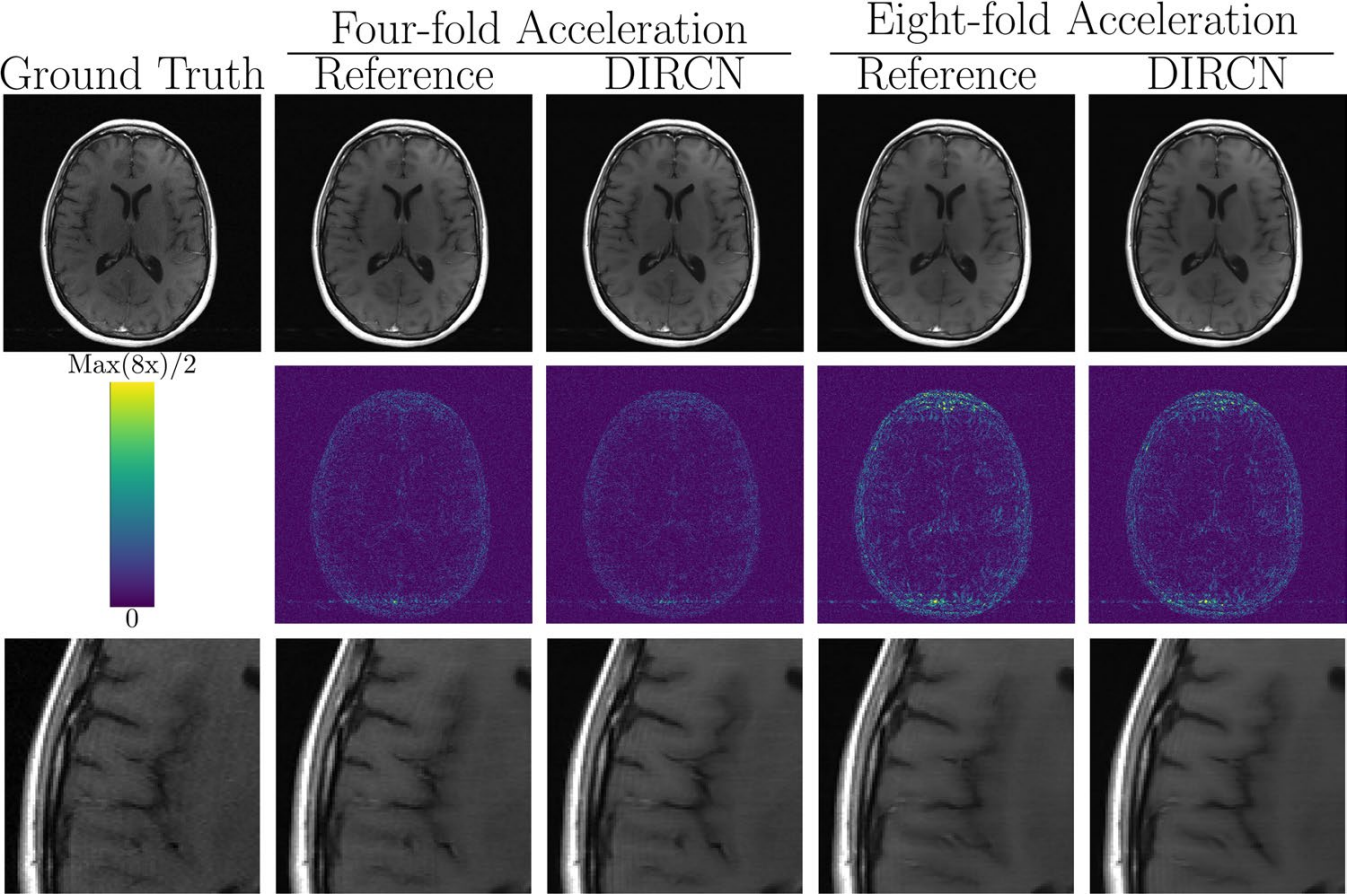
A representative example of a T1-weighted reconstruction with the reference model and DIRCN for four- and eightfold acceleration. This includes their respective reconstructions and the corresponding error map (absolute difference) between the fully sampled image and the reconstruction. The colormap goes between 0 and half the maximum error for eightfold acceleration to emphasize visual difference. The bottom images are a region of interest where slight improvement between the reference and DIRCN can be seen at close inspection

**Fig. 6:**
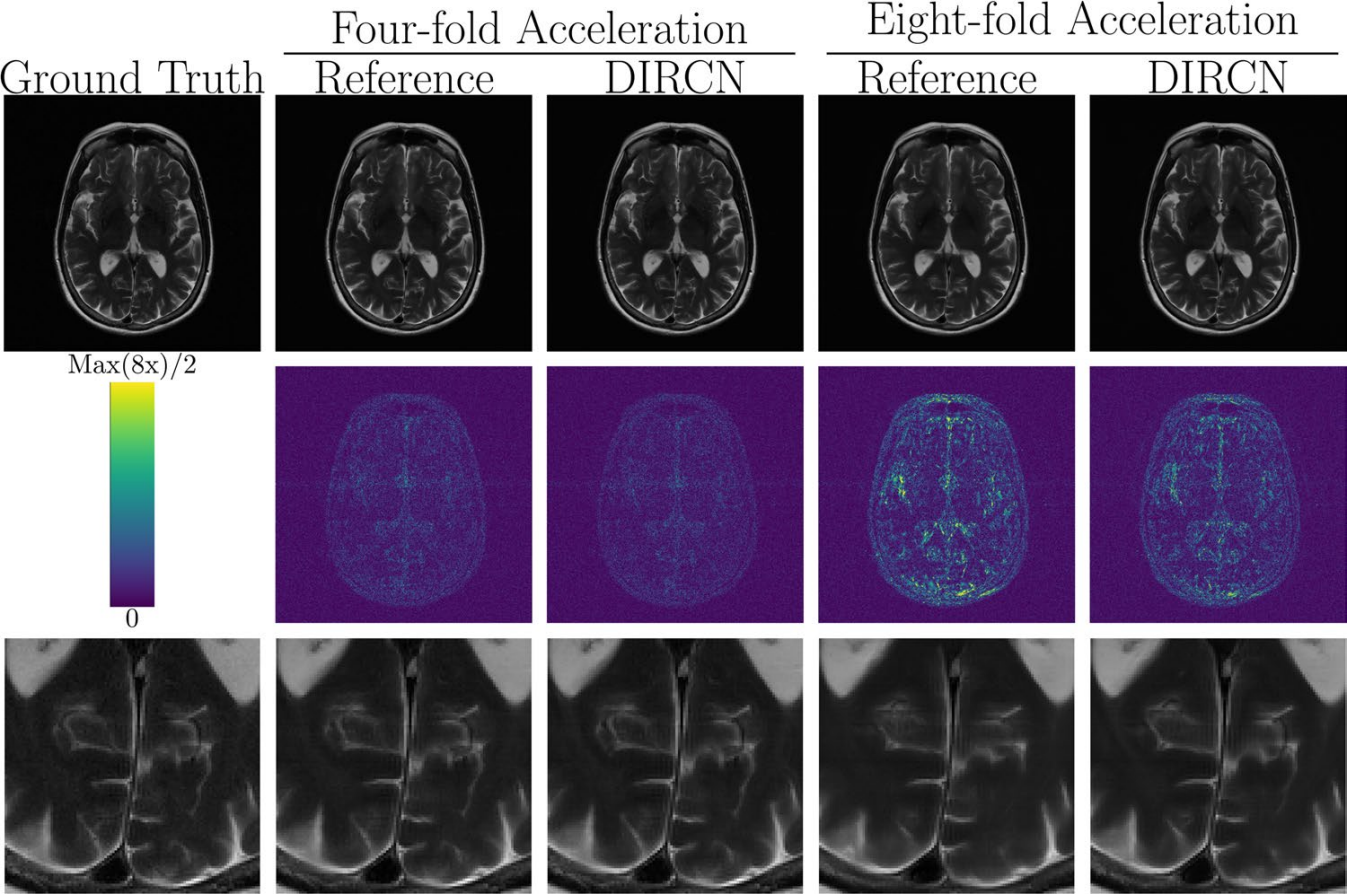
A representative example of a T2-weighted reconstruction with the reference model and DIRCN for four- and eightfold acceleration. This includes their respective reconstructions and the corresponding error map (absolute difference) between the fully sampled image and the reconstruction. The colormap goes between 0 and half the maximum error for eightfold acceleration to emphasize visual difference. The bottom images are a region of interest where differences between the reference model and DIRCN reconstructions for eightfold acceleration can be seen

**Fig. 7:**
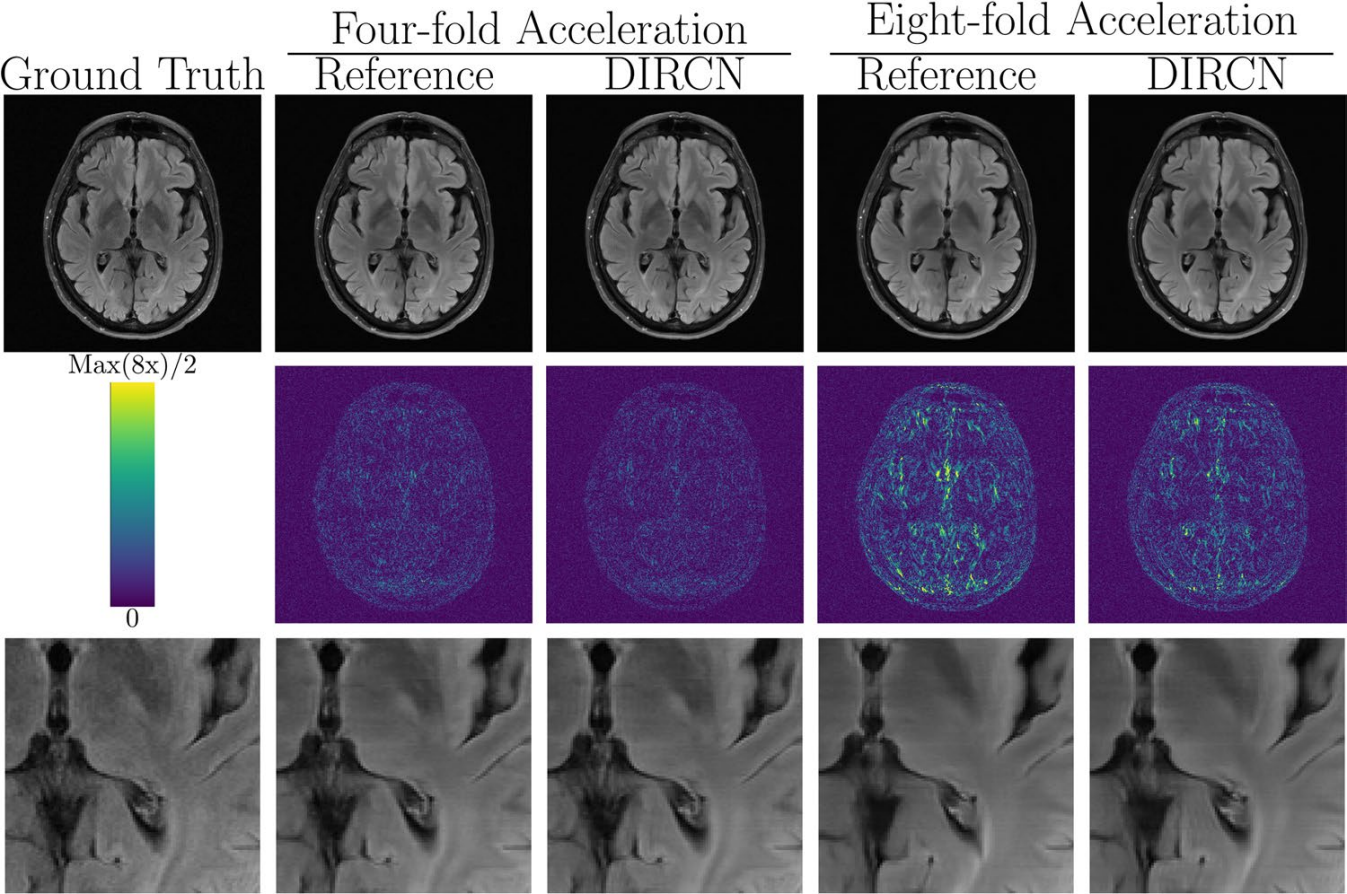
A representative example of a FLAIR reconstruction with the reference model and DIRCN for four- and eightfold acceleration. This includes their respective reconstructions and the corresponding error map (absolute difference) between the fully sampled image and the reconstruction. The colormap goes between 0 and half the maximum error for eightfold acceleration to emphasize visual difference. The bottom images are a region of interest where one can see an erroneous reconstruction for eightfold acceleration for both models

**Fig. 8:**
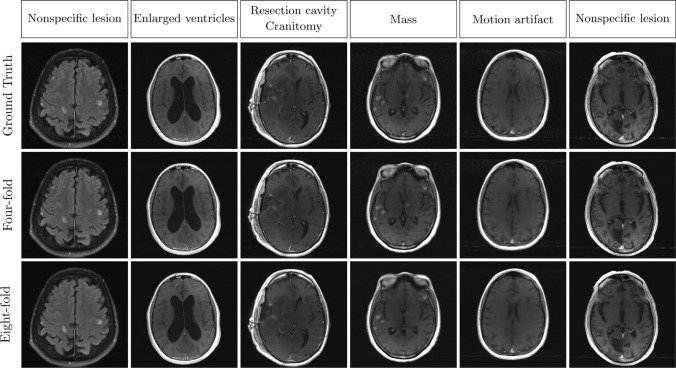
Different brain pathologies for four- and eightfold acceleration reconstructed with the DIRCN model. The pathological annotations are credited the fastMRI + initiative [43], and the images were selected at random from the test dataset from the labels selected above

Figures 5, 6, and 7 show representative reconstructions of magnitude T1-weighted, T2-weighted, and FLAIR images with their respective absolute error for the reference and DIRCN. The images were randomly selected from the reconstructions with a SSIM close to the mean SSIM reported in Table 1. A visual decrease in the absolute error between the reference model and DIRCN can be observed, especially for the eightfold accelerated images. Typically, DIRCN produces reconstructions that are closer to that of the ground truth image, as can be seen from the error maps. One such example can be discerned from the error map of the eightfold accelerated T2-weighted images. For the eightfold accelerated images, DIRCN show visible artifacts, but still outperforms the reference model for the same acceleration. Reconstructions with the DIRCN model for a variety of pathologies is shown in Fig. 8 for four- and eightfold acceleration with the corresponding ground truth; the pathology annotations are credited the fastMRI + initiative [43]. We note that the trained model can generalize to a variety of different pathologies.

**Fig. 9:**
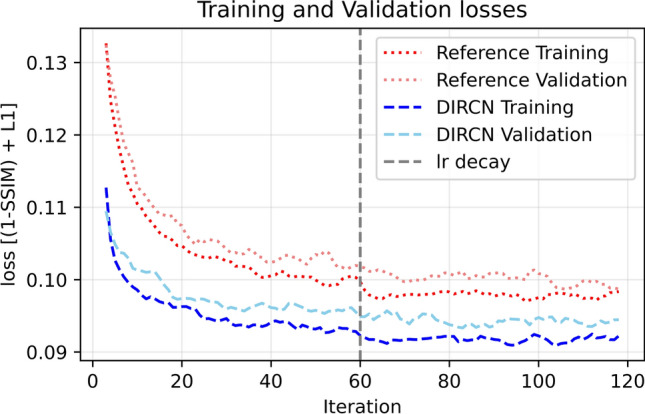
The training and validation losses for the reference and DIRCN for 120 iterations. The plotted losses are mean losses for a 5-point sliding window starting at iteration 3 and ending at iteration 117

**Fig. 10:**
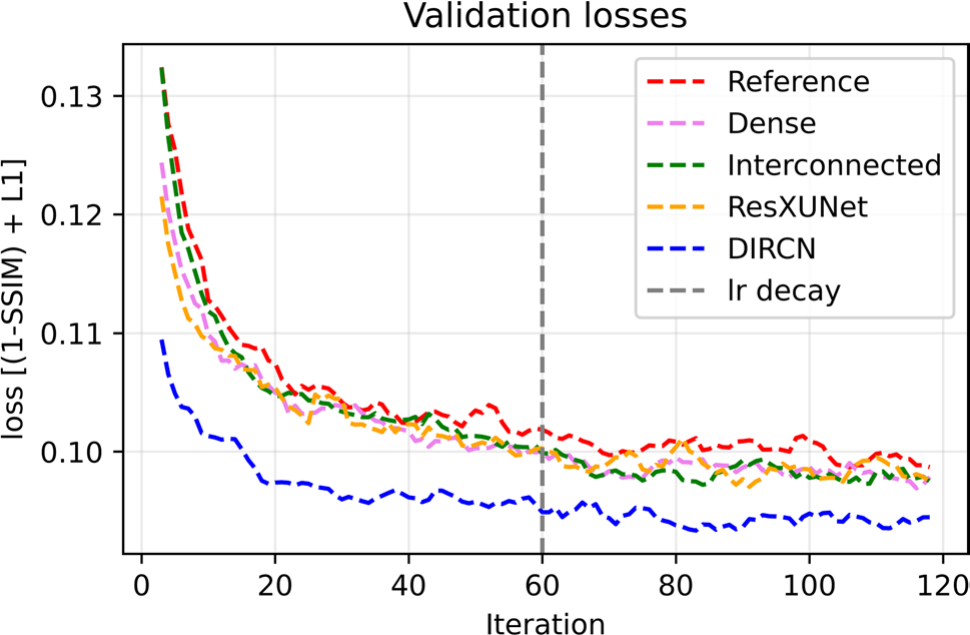
The validation losses for all network configurations for 120 iterations. The plotted losses are mean losses for a 5-point sliding window starting at iteration 3 and ending at iteration 117

The training and validation losses for the reference model and DIRCN are plotted in Fig. 9, and the validation loss for all network configurations are plotted in Fig. 10. A difference in convergence can be seen across the different configurations, and the dense and residual configurations have a high initial convergence. The configuration with interconnections had a similar initial convergence to that of the reference model. However, the convergence rate increased after an initial phase. No major sign of overfitting can be discerned from Fig. 9; there is, however, a slight divergence between the training and validation loss. This divergence was observed for all model configurations, but slightly more pronounced for DIRCN when compared to the reference model.

**Fig. 11:**
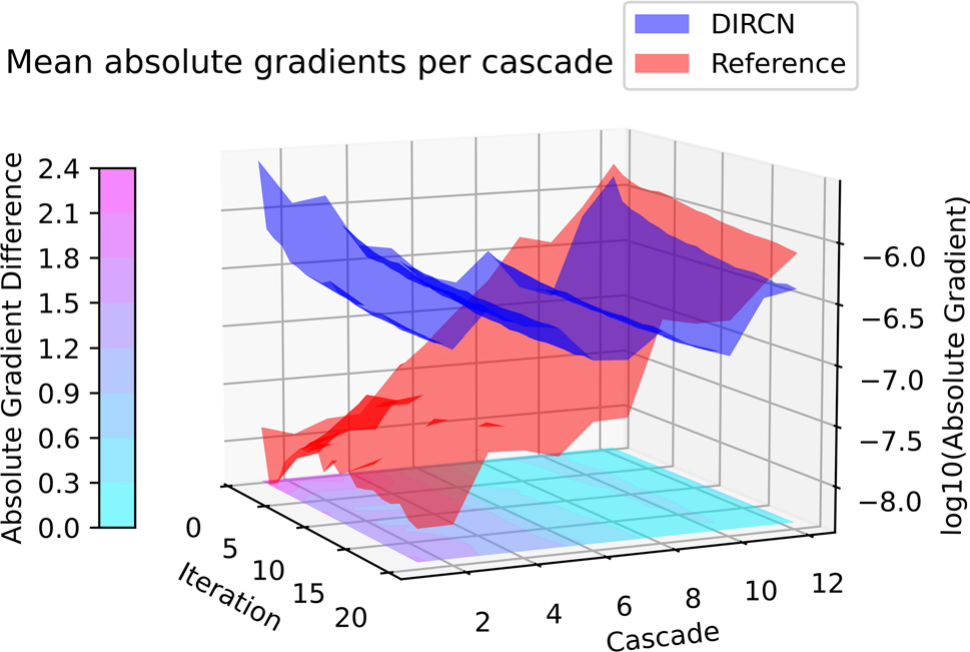
The logarithm with base 10 of the mean gradients for every learnable parameter per cascade for the first 20 iterations. A contour plot of the absolute difference between the logarithm of the mean gradients between the networks is embedded into the 3D visualization with the corresponding colorbar. Mean gradient values were $\sim {10}^{-6}$ for the DIRCN, whereas the reference model had mean absolute gradient values ranging ${10}^{-6}-{10}^{-9}$ depending on the cascade

The logarithm for the mean absolute gradient values per cascade for the 20 first iterations is plotted in Fig. 11. In the first cascades, the mean absolute gradient is a magnitude of 100 larger in DIRCN when compared to the reference model. Besides the larger gradient values, the mean absolute gradient is more stable throughout the network in contrast to the reference model, where the difference between the first and last cascades is in the order of 100 times less.

## Discussion

The DIRCN showed superior metrical results compared to the reference model, with the error maps being closer to that of the ground truth image, and the SSIM and NMSE showing over 10% increase for both acceleration factors. Although the reconstructions are closer to that of the ground truth, it is difficult to discern any visual difference on the magnitude images, since the reconstructions of the reference model are state-of-the-art.

Similar to a previous work [19], this work showed an improved performance with the addition of input-level dense connections. In addition, the dense connections had no noticeable inference time or memory overhead when compared to the reference model. The proposed interconnections showed a similar increase in performance as the dense connections, with no noticeable increase in inference time or memory overhead. The minor increase in the number of parameters came from an increase in the number of incoming channels in the concatenation operation. Unlike the dense connections, the implementation of the interconnections can be modified within the network to further improve performance. A possible improvement could be to use attention similar to attention U-Net [44]. In addition, interconnections could be implemented to connect additional subsequent sub-networks, which may further improve the overall performance. However, this was opted against to avoid additional computational bottlenecks, and was outside the scope of this study.

The ResXUNet model used in this study achieved improved performance in comparison to the reference model. However, this improved performance introduces additional computational complexity, which translates to increased memory consumption and inference time. Because of these overheads, additional research into the most suitable sub-network architecture is necessary. Nonetheless, the increase in performance may warrant the additional computational complexity. Future studies should be performed to find an ideal tradeoff between computational overhead and overall performance increase.

The results shown in this work may further improve through more optimized training strategies, such as parallelization with a larger batch size than one, data augmentation, or a different optimizer. Architectural improvements could include spatial attention through the convolutional block attention module [45] or vision transformer based methods that have shown great promise in MRI synthesis and reconstruction [46, 47]. Besides architectural additions, length scaling by increasing the number of cascades could further improve the performance as has been shown in previous works for iterative MRI reconstruction [48–50]. In addition, length scaling could be combined with deep supervision to further emphasize gradient flow [51] to further enhance the trend shown in Fig. 11. Additionally, separate training for four- and eightfold acceleration and extended training time could further improve the results, and potentially coined with a transfer learning-based approach [52]. Besides architectural and training-wise modifications, the DIRCN does not perform reconstruction in the frequency domain, while studies have suggested dual domain reconstruction may improve the resulting reconstruction [17, 22].

The study has limitations in that the model has only been trained on retrospective public domain data. As such, it is necessary to further test the model on clinically valid prospective data on in-house MRI systems. In addition, in this work, the effects of the undersampling scheme on the model extensions were not evaluated. However, as the enhancements are of architectural nature, it is not unreasonable to assume that other undersampling schemes may benefit from the proposed enhancements. In addition, we note that the reference used for benchmarking purposes deviates slightly from the end-to-end variational network [6] with respect to the data consistency method. Nonetheless, during an initial testing phase, both methods showed similar performance. Lastly, different model configurations with respect to the number of channels and cascades were not tested. This includes weight sharing that was deemed redundant since the NYU fastMRI neuro dataset contains a large amount of training data.

## Conclusion

Inspired by the end-to-end variational network, multiple architectural improvements were tested and evaluated. Experimental result demonstrates that input-level dense connections, a modified convolutional sub-network, and interconnections (long-range skip connections) improved the quality of the reconstructed images for both four- and eightfold acceleration. Our findings suggest the importance of gradient flow and shared information between cascades for MRI reconstruction networks. The proposed DIRCN attains improved results over the reference model, and more fine structures were visibly preserved for eightfold acceleration in the reconstructions. It is shown that simple alterations and additions to enhancing the cascading framework can improve the overall quality of the reconstruction.

## Footnotes

1 https://github.com/JonOttesen/DIRCN.

2 Overline denotes the complex conjugate, not complex transpose.

3 Superscript denote the cascade number.
